# *Pax6* Expression Is Sufficient to Induce a Neurogenic Fate in Glial Progenitors of the Neonatal Subventricular Zone

**DOI:** 10.1371/journal.pone.0020894

**Published:** 2011-06-17

**Authors:** Eun Sook Jang, James E. Goldman

**Affiliations:** 1 Integrated CMBS and Pathology Graduate Programs, Columbia University, New York, New York, United States of America; 2 Department of Pathology and Cell Biology, Columbia University, New York, New York, United States of America; University of Dayton, United States of America

## Abstract

**Background:**

The forebrain subventricular zone (SVZ) of neonatal mammals contains a large, heterogeneous population of migratory and proliferating precursors of interneurons and glia. These cell types are produced in large numbers in the immediate postnatal period, the glioblasts populating the hemispheres with astrocytes and oligodendrocytes, the neuroblasts migrating to the olfactory bulb to become interneurons. How cell fate decisions are determined or stabilized in this mixed population is not clear, although previous studies indicate the importance of two transcription factors, Pax6 in neurons and Olig2 in glia, and suggest there may be reciprocal repression between these genes.

**Methodology/Principal Findings:**

In examining the SVZ of neonatal mouse and rat brain, we find that the very large majority of SVZ cells express either Pax6 or Olig2, but few express both. We have used *in vivo* retro- and lenti-virus injections into the neonatal SVZ and *in vitro* gene transfer to demonstrate that *pax6* over-expression is sufficient to down-regulate *olig2* and to promote a neuronal lineage development and migration pattern in *olig2*-expressing cells. Furthermore, we provide evidence that Pax6 binds to the *olig2* promoter and that an HEB sequence in the promoter is required for the Pax6 repression of *olig2* transcription. Lastly, we constructed a lentivirus to target *olig2*-expressing cells in the SVZ to trace their fates, and found that the very large majority developed into glia.

**Conclusions/Significance:**

We provide evidence for a direct repression of *olig2* by Pax6. Since SVZ cells can display developmental plasticity *in vitro*, the cross-repression promotes a stabilization of cell fates. This repression may be critical in a germinal zone in which immature cells are highly migratory and are not organized into an epithelium.

## Introduction

During the first few postnatal weeks of rodent forebrain development, the subventricular zone (SVZ) generates both neurons and glia [1]–[5].OHHO This is a region of highly migratory and proliferative cells, glioblasts populating the hemispheres with astrocytes and oligodendrocytes, and neuroblasts migrating to the olfactory bulb, where they become interneurons. In culture SVZ cells manifest plasticity of fate, since clonal studies of SVZ cells *in vitro* show that individual cells can give rise to both neurons and glia [6]. One would imagine, however, that these highly migratory cells in the SVZ [7], all exposed to a similar extracellular environment, would require some mechanism to restrict fate plasticity *in vivo*. The mechanisms of cell fate determination and stabilization in this complex region at this time are not well understood.

In this study, we have focused on two transcription factors, Pax6 and Olig2 in the neonatal forebrain SVZ. Pax6 has both homeo- (HD) and paired- (PD) DNA binding domains [8], [9], which contribute to transcriptional activation (αA-crystallin) [10] or repression (MMP2, Pax2, and αB1-crystallin) [11]–[13], depending on the cellular context and promotes neurogenic cell fates during development. [14]–[16]. The basic helix-loop-helix transcription factor, Olig2, functions as a transcriptional repressor by dimerizing with E proteins [17]–[19]. During forebrain development, Olig2 appears to be critical for glial cell fate determination. Recently, we found that *olig2* specifies neonatal SVZ cells to produce glial precursors, but not neurons, and suggested that *olig2* expression was sufficient to change a neurogenic to a gliogenic fate in this population [20].

There is genetic evidence of reciprocal interactions between Pax6 and Olig2 during spinal cord and forebrain development. Thus, the *pax6*-null mouse shows premature glial specification by the ectopic expression of olig1/2 in spinal cord [21] and the gene expression pattern of *pax6*-deficient E15 mouse cortex or ganglionic eminence shows increased expression of *olig2* [22]. In the E14 *pax6* mutant cortex, the normal, ventral *olig2* expression expands dorsally into the ventricular zone in which *pax6* is normally expressed [16] and in an *olig1/2* double mutant or *olig2* loss of function mouse , the *pax6*-expressing spinal cord domain V2 expands ventrally to occupy the place of the pMN domain, which normally expresses *olig2* [18], [23]. Antagonizing Olig2 function leads to the upregulation of Pax6 in an acute lesion that activates Olig2 expression [24]. Finally, overexpressing *olig2* in Pax6+ neuroblasts of the adult rodent SVZ converts cells to Olig2+/Pax6- glia that migrate into the overlying corpus callosum [25]. The mechanism(s) underlying this apparent reciprocal expression remains unknown.

Here, we have examined Pax6 and Olig2 in the neonatal forebrain SVZ and find individual cells contain Pax6 or Olig2 but rarely both. Furthermore, we find that Pax6 represses *olig2* expression in this population, leading to a downregulation of *olig2* and a loss of Olig2 protein. The overexpression of *pax6* is sufficient, in the neonatal SVZ, to repress a gliogenic fate choice and to promote a neurogenic choice. Finally, we find that the Olig2+ population in the neonatal SVZ develop almost entirely into glia, suggesting that the olig2 and pax6 populations are not only distinct, but have different fates, and that a cross-repression between these two factors is important in stabilizing a neuronal or glial cell fate.

## Results

### Pax6 and Olig2 have mutually exclusive expression patterns in the neonatal forebrain SVZ

**Figure 1:**
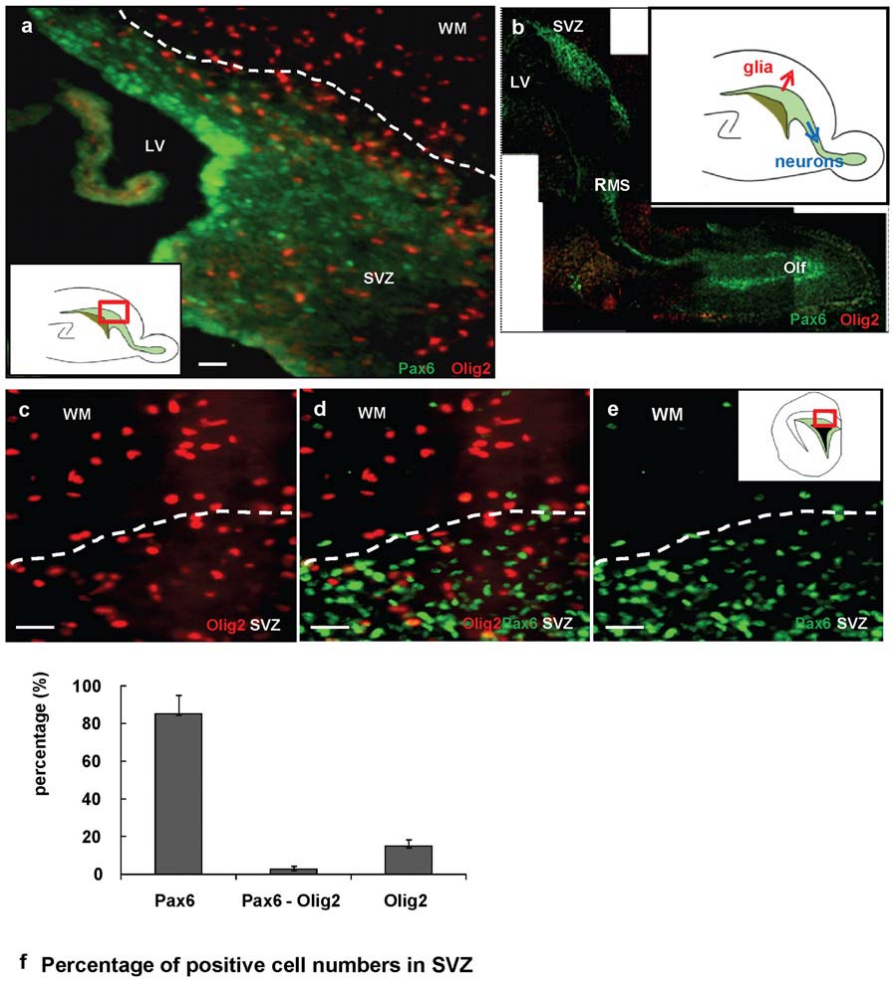
Mutual exclusive expression of the transcription factors Pax6 and Olig2 in the postnatal SVZ. (**a**) Pax6+ (green) and Olig2+ (red) visualized in a parasagittal plane of P4 mouse SVZ. (**b**) Pax6+ (green) and Olig2+ (red) in a parasagittal section of P2 rat brain through the RMS. Inset depicts the radial migration of glial progenitors and the rostral migration of neuronal progenitors. (**c**–**e**) Cells in a coronal section of P2 rat forebrain. (**c**) Olig2 (red), (**d**) merged, and (**e**) Pax6 (green). Inset (**a**), (**e**) depicts the area of photomicrographs shown. (**f**) Histogram summarizing percentage of Pax6+ or Olig2+ cells in P4 mouse SVZ. Quantified distributions of cells presented as a percentage of infected cells per brain located in each area (mean ± SEM). SVZ; subventricular zone, LV; lateral ventricle, RMS; rostral migratory stream, Olf; olfactory bulb, WM; white matter. Scale bars: 50 µm.

To characterize the spatial expression patterns of Pax6 and Olig2 in the neonatal mouse and rat forebrain SVZ, we performed double immunofluorescence analysis on sections of P2–4 SVZ and RMS (**Fig. 1** **, and Fig. S1**). In both areas and in both species the cellular localizations of Pax6 and Olig2 appeared almost entirely mutually exclusive (**Fig. 1a**–**e**). Pax6 was observed in the SVZ and RMS (**Fig. 1b**). Within the SVZ, there was a higher density of Pax6+ cells close to the ventricular surface. Olig2+ cells were found in the SVZ, where they tended to concentrate in the center and periphery of the region, and in tissue surrounding the RMS, but few appeared in the RMS itself (**Fig. 1a, b**) [20]. There were many Olig2+ cells in the overlying white matter (**Fig. 1c**–**e**), as expected at this time of vigorous gliogenesis. Pax6+/Olig2+ cells were rare (less than 3% of total Pax6+ and Olig2+ cells) (**Fig. 1f**). We compared Pax6 and Olig2 immunostaining with the nuclear marker, DAPI, at this coronal level (at the level of septal nuclei, where we performed viral injections, see below). We found that about 85% of the SVZ cell nuclei were Pax6+ and about 15% were Olig2+. Thus, the large majority of SVZ cells at this age expressed either one or the other of these factors (**Fig. 1f**). The small numbers of non-Olig2+ or non-Pax6+ cells mostly expressed markers of endothelial cells or microglia (data not shown).

To investigate the identity of the Pax6+ and Olig2+ cells, we performed co-immunostaining with antibodies against cell type specific markers (**Fig. S2**). Almost half of the Pax6+ cells were co-stained with the neuronal ß-tubulin marker, TuJ1 (**Fig. S2g, i**). Because of the large amount of TuJ1 signal in this region of the forebrain, we thought we could obtain better cellular resolution with isolated cells, and so we isolated SVZ cells from P2 mouse forebrain and immunostained them for Pax6+, TuJ1+, and olig2 after only 1 hour. About 50% of the Pax6+ cells were also TuJ1+ (data not shown), matching the result from tissue sections, and double staining for Olig2 and TuJ1+ showed no overlap. A substantial percentage of the Olig2+ cells were positive for the NG2 chondroitin sulfate proteoglycan (48% of Olig2+ cells) (**Fig. S2k, l**) or ZebrinII (18% of Olig2+ cells) (**Fig. S2b, c**). Almost 60% of Pax6+ (**Fig. S2d, f**) and fewer than 5% of Olig2+ cells (**Fig. S2e, f**) were positive for RC2, a radial glial cell marker. Thus, in general, Pax6 localization correlates with early neuronal development. Olig2 localization is different, although more complex at this age, since both ZebrinII and RC2 are expressed by radial glia and ZebrinII is also found in GFAP+ cells of the neonatal SVZ as well as in developing astrocytes [25], [26], so that neither of these markers necessarily implies a commitment to a glial lineage.

### SVZ progenitors overexpressing pax6 differentiate along a neuronal lineage *in vivo*

Previous work in our laboratory showed that the constitutive expression of olig2 by retroviral infection into neonatal SVZ cells caused the cells to migrate radially into white matter and cortex and to differentiate into glia [20]. In contrast, control retrovirus-infected SVZ cells displayed a mixed pattern: some migrated radially to become glia, while others migrated anteriorly along the RMS to become olfactory bulb interneurons (**Fig. S3**) [7], [20]. We now asked if the expression of pax6 was sufficient to influence SVZ cell fate in vivo, by performing injections of retroviruses containing pax6 or pax6(5a)-R128C into the neonatal SVZ to ask if the expression of these genes alters the fates and migratory patterns of SVZ cells.

**Figure 2:**
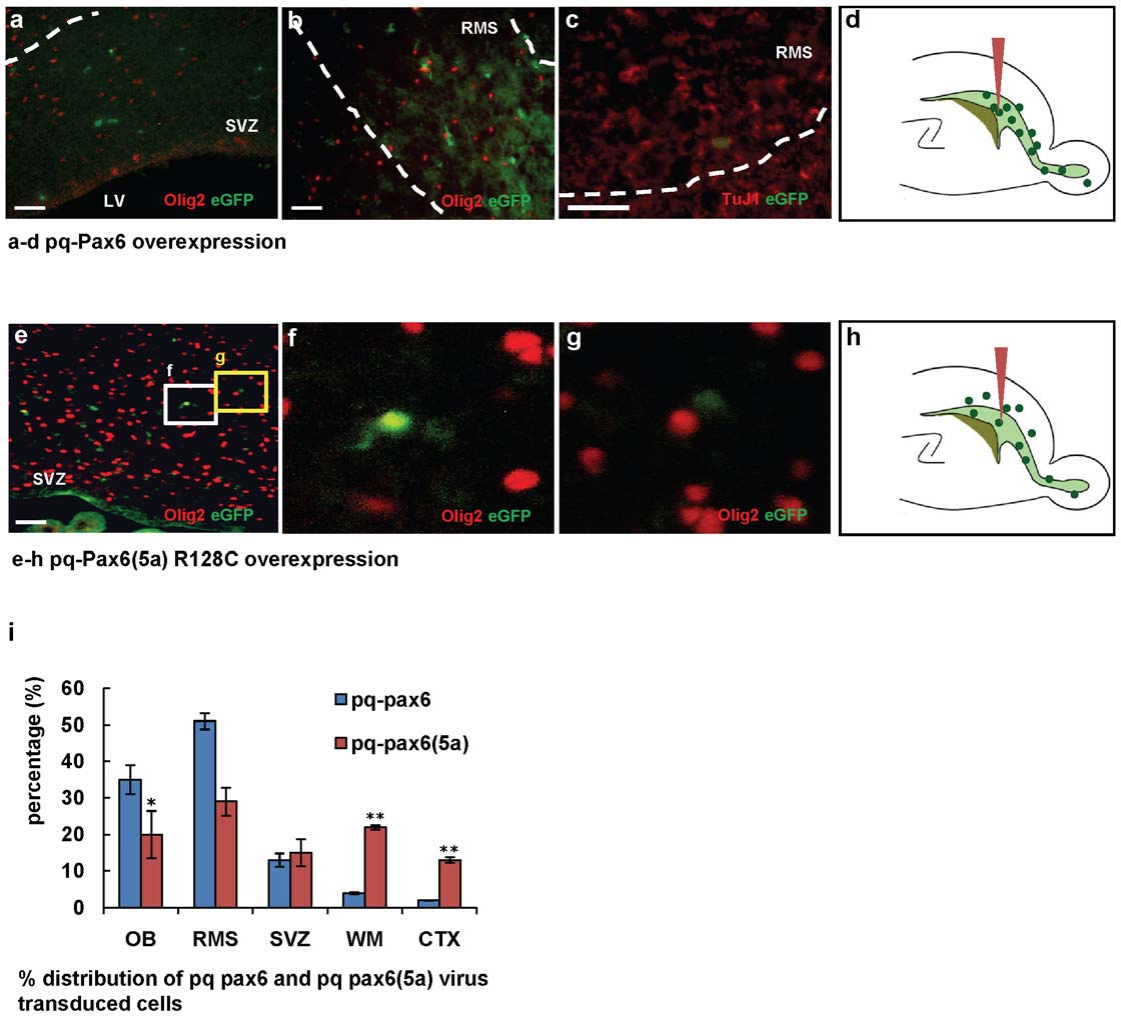
SVZ cells infected with retroviral-expressed *pax6* differentiate along a neuronal lineage *in vivo*. Retroviruses were injected into the SVZ of P2 rat brains (n = 7 for each condition) analyzed 4 d later. (**a**–**d**) *pax6-IRES-eGFP* infected cells migrated into the RMS. All eGFP+ cells are Olig2- in SVZ (**a**) and RMS (**b**) and express TuJ1 in the RMS (**c**). (**d**) Distribution of cells. (**e**–**h**) *pax6(5a) R128C-IRES-eGFP* infected cells exhibit a normal (mixed glial and neuronal) migration pattern. (**e**) Olig2+/eGFP+ or Olig2-/eGFP+ infected cells are observed in SVZ. (**f**) White box shows Olig2+/eGFP+ cell. (**g**) Yellow box shows Olig2-/eGFP+ cells. (**h**) Distribution of cells. (**i**) Histogram analysis of the effects of *pax6* and *pax6(5a) R128C* infection on migratory behavior, presented as the percentage of infected cells per brain located in each area (mean ± SEM). ^*^, by student's *t* test *p*<0.05,^ **^, *p*<0.01.Scale bars, 50 µm.

First, immunostaining of P2 rat brain SVZ with the proliferation marker, Ki67, showed that 71% of Pax6+ and 67% of Olig2+ SVZ cells are Ki67+. Thus, the large majority of these cells are cycling and thus amenable to retroviral insertion (**Fig. S4a**, **b**) and there does not appear to be preferential cycling of one population. To investigate whether *pax6* expression induces a neuronal type migration and differentiation, we generated *pax6-IRES-eGFP* and *pax6(5a)-R128C-IRES-eGFP* expressing retroviruses, the latter representing a DNA binding mutant (30), (**Fig. 2** **k,l**) and injected them into the P2/3 rat SVZ. At 4dpi of the wild type *pax6* vector, over 90% of the eGFP+ cells were found in the SVZ, RMS, and olfactory bulb (**Fig. 2a**–**d**). eGFP+ cells in the SVZ and RMS did not show detectable Olig2 (**Fig. 2a,b**). Most of the *pax6* infected cells that had migrated into the RMS expressed the neuronal marker, TuJ1 (**Fig. 2c**). Therefore, SVZ progenitors constitutively expressing *pax6* showed a neuroblast-like, tangential migration pattern and early neuronal differentiation. In contrast, SVZ cells expressing *pax6(5a)-R128C,* exhibited a normal migration pattern, both tangential and radial, into the RMS, white matter and cortex (**Fig. 2e**–**h**). Almost 18% of the eGFP+ cells in the SVZ and white matter were also Olig2+ (**Fig. 2e,f**). For quantification of the relative distributions of infected cells, we examined three comparable parasagittal sections from 4 brains infected with the *pax6* virus and 4 brains infected with the *pax6(5a) R128C* at 4dpi (**Fig. 2i, j**). We observed the same results after injections of these retroviruses into P3–4 mouse forebrain SVZ (not shown).

The retroviral data suggest that Pax6 promotes a neuronal fate in this population. None of the cells expressing retroviral-mediated Pax6 was positive for Olig2. Immunostaining for the cell death marker, cleaved caspase3, did not reveal any significant cell loss resulting from vector injection (**Fig. S6a**, **b**). Thus we have no evidence that overexpression of any of these viruses affected the cell fates of the population by selectively killing immature glia.

### *Pax6* targeted to Olig2+ cells *in vivo* and in culture results in lower Olig2 levels and a neuronal fate

Because the SVZ contains a heterogeneous population of dividing cells, retroviruses label both neuroblasts and glioblasts [7]. Despite this heterogeneity, we observed dramatic shifts in cell fates after *pax6* expression by retroviruses (or *olig2* expression by retroviruses, [20]), suggesting that the expression of each transcription factor was sufficient to produce a loss of the other factor, followed by the acquisition of glial or neuronal characteristics. In addition, we used two strategies to target transcription factor expression to more defined sets of SVZ cells. In the first, we took advantage of the *olig2*-*Cre* mouse to express *pax6 in vivo* specifically in Olig2+ cells. In the second, we sorted SVZ cells from *olig2-GFP* mice to demonstrate directly that expression of *pax6* leads to a loss of Olig2.

**Figure 3:**
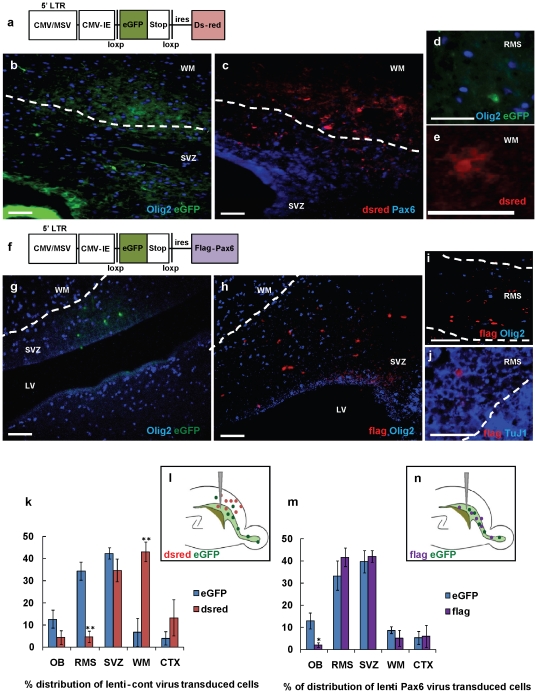
Down regulation of Olig2 by lentiviral Pax6 overexpression promotes tangential migration and neuronal differentiation. The SVZ of P3/P4 *olig2*-*Cre* mouse was stereotactically injected with lentiviruses expressing *loxp-GFP-stop-loxp-dsred* (control) (n = 5), or *loxp-GFP-stop-loxp-flag-pax6* (Pax6-lenti) (n = 6) and brains analyzed 4 days later. (a, f) Schematics of control (a) and Pax6-lenti (f) constructs. (b–e) eGFP+ cells infected by control-lentivirus, which were Olig2- at infection, migrated tangentially, whereas dsred+ cells, which were Olig2+, showed radial migration. (b) eGFP+ cells were Olig2- in SVZ. (c) Ds-red+ cells located in WM, SVZ and Ctx, are Pax6-. (d) eGFP+ cells were Olig2- in RMS. (e) Ds-red+ cells showed glial morphology in WM. (g–j) Both eGFP and flag-tagged Pax6 expressing cells by Pax6-lenti virus migrated tangentially. (g) eGFP+ cells were Olig2- in SVZ. Flag+ cells, which were Olig2+ at infection, were Olig2- in SVZ (h) and RMS (i). (k, m) Histogram analysis of the effects of control (j) or Pax6-lentivirus (l) on migration. Quantified distributions of cells presented as a percentage of infected cells per brain located in each area (mean ± SEM). ^*^, by student's *t* test *p*<0.05,^ **^, *p*<0.01. (l, n) Schematic distribution of SVZ cells expressing control (l) or Pax6 (n) and site of injection. Scale bars, 50 µm.

For the *in vivo* studies, we generated a lentiviral vector that contains a *loxp-GFP-stop-loxp-flag-pax6* sequence (**Fig. 3f**) and injected this virus into the neonatal SVZ of *olig2*-*Cre* mice. (n = 5) For a control, we used a dual reporter virus that contains *Ds-red* instead of the *flag-Pax6* (**Fig. 3a**) (n = 4). If the *pax6* lentiviral vector incorporates into an *olig2*-*Cre* positive cell, then the e*GFP-stop* sequence will be excised and the cells will lose eGFP and begin to express the flag-Pax6. If the vector incorporates into an *olig2*-*Cre* negative cell, then the cell will remain eGFP+ and not express flag-Pax6. In case of the control lentivirus infection, only the *olig2*-Cre positive cells will express Ds-red, whereas the *olig2*-*Cre* negative cells will express eGFP.

First, the control *floxed eGFP-stop-Ds-red* expressing lentivirus was injected into the SVZ of P3/4 *olig2*-*Cre* mouse brains, and the tissues analyzed 4 days later. We found eGFP+ cells mostly in the SVZ and the RMS (∼88%) (**Fig. 3b, d, k**), whereas Ds-red+ cells were localized to the SVZ (∼35%) and overlying white matter and cerebral cortex (∼56%) (**Fig. 3c, e, k**). All the eGFP+ cells were Olig2- (**Fig. 3b, d**), while all the Ds-red+ cells were Olig2+ (**Fig. S5a**–**c**) and Pax6- (**Fig. 3c**). The Ds-red+ cells showed glial cell morphologies (**Fig. 3e**). We found a few eGFP+ cells in the white matter and lower cortex (∼10%of total eGFP+ cells, data not shown), which are extremely rare with retroviral injections, in our experience (but see [28], which reported interneurons generated from dividing SVZ cells in the newborn mouse frontal cortex). These lentivirus-labeled cells may not have been proliferating at the time of injection, since, unlike the retrovirus, the lentiviral backbone can be incorporated and expressed in non-proliferating cells. Finding these cells with neuronal characteristics correlates with our unpublished data with other lentivirus injections into the neonatal SVZ (Personal communication, R. Ventura). We also found rare Ds-red+ cells in the RMS (**Fig. 3k, l**). These cells might represent neuroblasts, or at least cells migrating as neuroblasts, that were generated from Olig2+ cells in the SVZ, but they were very rare.

Pups were then injected with the *floxed eGFP-stop-flag-pax6* lentivirus, and cells visualized by eGFP and/or flag expression 4 days after injection. More than 85%of eGFP+ cells were localized to the SVZ, RMS, and olfactory bulb, as expected (**Fig. 3g, m**). Flag+/Pax6+ cells, which were Olig2+ at the time of infection, migrated tangentially and were also localized to the SVZ (∼42%), RMS (∼41%) and olfactory bulb (∼2%) (**Fig. 3m,n**). The flag+ cells were Olig2- in SVZ (**Fig. 3h**) and RMS (**Fig. 3i**). In addition, most of the flag+/Pax6+ cells in the RMS and olfactory bulb also expressed the neuronal marker TuJ1 (**Fig. 3j** **, Fig. S5d**). These observations strongly suggest that the expression of *pax6* in an Olig2+ cell can alter the cell lineage from glial to neuronal. No increased cell death was observed in *olig2*-Cre mice injected with control or *flag-pax6* viruses (**Fig. S6c**–**f**). For quantification of the relative distributions of infected cells, we used a sampling of three comparable parasagittal sections from 4 brains injected with control lentivirus and 3 brains injected with *pax6* lentivirus. (**Fig. 3k, m**)

**Figure 4:**
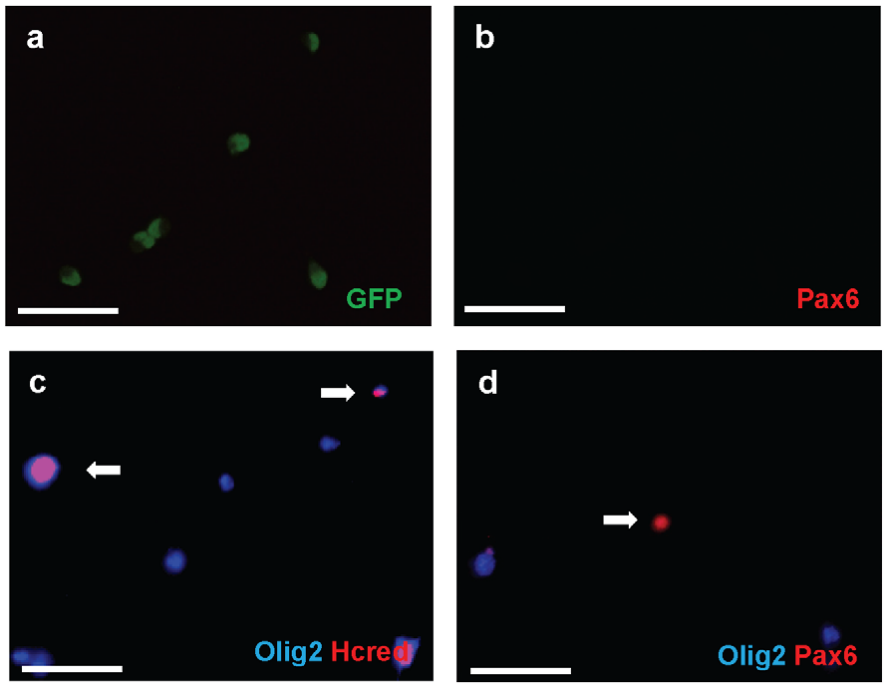
*In vitro* overexpression of Pax6 inhibits cell intrinsic expression of Olig2. The *olig2*-GFP mouse SVZ was manually dissected from P4 pups. Tissue was washed in PBS, then incubated to 0.05%trypsin/0.53 mM EDTA (Invitrogen) at 37°C for 10 min and triturated. Trypsin was neutralized with PBS with 2%FBS. Neutralized cells were filtered and separated with 4%glucose PBS solution. Cell sorting was performed in the Columbia FACS facility using a modified triple laser FACS instrument and Summit software (DAKO) or DIVA software (BD Biosciences). The FlowJo program (Tree Star) was used for flow cytometry data analysis. Sorted Olig2-GFP cells were attached on glass slides and cultured in B1O4 conditioned medium (Hunter and Bottenstein, 1990; Hunter and Bottenstein, 1991) mixed 1∶3 with DMEM and then transfected with either *pax6* or control *eGFP* expression constructs. Infected cells were kept in culture with basal media (DMEM, N2, T3, and penicillin/streptomycin/amphotericin) for 2d and then fixed in 4%paraformaldehyde, stained and analyzed by fluorescence microscopy. (**a**, **b**) After sorting, Olig2-eGFP positive cells were plated, incubated 7 days and immunostained with antibodies against Pax6. Virtually all of Olig2-eGFP+ cells (**a**) are Pax6-(**b**). eGFP immunostaining in green, Pax6 in red. (**c**) Sorted Olig2-eGFP cells were transfected with control Hcred vector. 48 hrs after transfection, immunostaining was done with anti-Olig2 antibody. Hcred immunostaining in red, Olig2 in Cy5. (**d**) Sorted Olig2-eGFP cells were transfected with the vector expressing flag-tagged wt Pax6. Each result was obtained by three independent experiments. Double immunostaining with anti-Olig2 and Pax6 antibodies demonstrates that transfected cells were Pax6+ but Olig2-. Olig2 immunostaining in Cy5, Pax6 in red. Scale bars, 50 µm.

In the second strategy, we FAC sorted SVZ cells from P4 *olig2-GFP* mice, cultured the cells, and analyzed them by subsequent immunofluorescence staining (**Fig. 4**). Immunostaining revealed that the Olig2-eGFP+ sorted cells did not contain detectable Pax6 even after 7 days in culture (**Fig. 4a, b**). Next, to determine whether *pax6* can repress endogenous Olig2 expression in these cells, we transfected the *flag-pax6* expression construct (*p3xFLAG-Pax6*) 2 days after plating the cells. Cultures were fixed and immunostained with anti-flag and anti-Olig2 antibodies 3 days later. We observed that the Pax6-flag+ cells showed very low or undetectable levels of Olig2 (**Fig. 4d**). Such a loss of Olig2 was not observed after cells were transfected with a control *HC-red* construct (**Fig. 4c**).

### *In Silico* approach identifies putative Pax6 binding sites on the promoter regions of *olig2*

**Figure 5:**
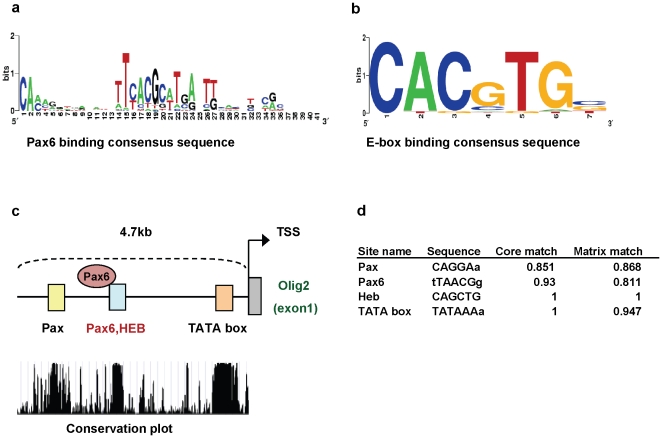
Predicted Pax6 binding sites on the *olig2* promoter region. (**a**, **b**) Graphical representations of consensus sequence for Pax6 (**a**) or E box (**b**) binding site, used as a matrix for prediction. (**c**) Schematic of predicted transcription factor binding sites of *olig2* promoter region. Colored boxes represent transcription factor binding sites. Pax6 binds to the blue Pax6 region. (**c**, **d**) Sites are selected after comparing the sequence conservation across 5 vertebrate species**.** Scores from the transcription factor binding site analysis for *pax6* gene promoter region used Match program in Transfac database software package.

One possible conclusion from the above studies is that Pax6 represses *olig2*. To investigate putative transcriptional interactions between the *olig2* promoter and Pax6, we first used an *in silico* approach to identify transcription factor binding sites in the 5′ upstream promoter sequences of *olig2*. Four effective transcription factor binding sites were predicted in the *olig2* promoter sequence, with above 90%identity across 5 vertebrate species **(** **Fig. 5c** **).** Two of them are Pax6 and HEB binding sites around 1.7 kb upstream of the transcription start site (TSS); the other is a generic Pax binding site (about 2.7 kb upstream of TSS), and the last is a TATA binding site, which is near the TSS (**Fig. 5c, d**).

### ChIP data demonstrates binding of Pax6 to *olig2* promoter regions *in vivo*

**Figure 6:**
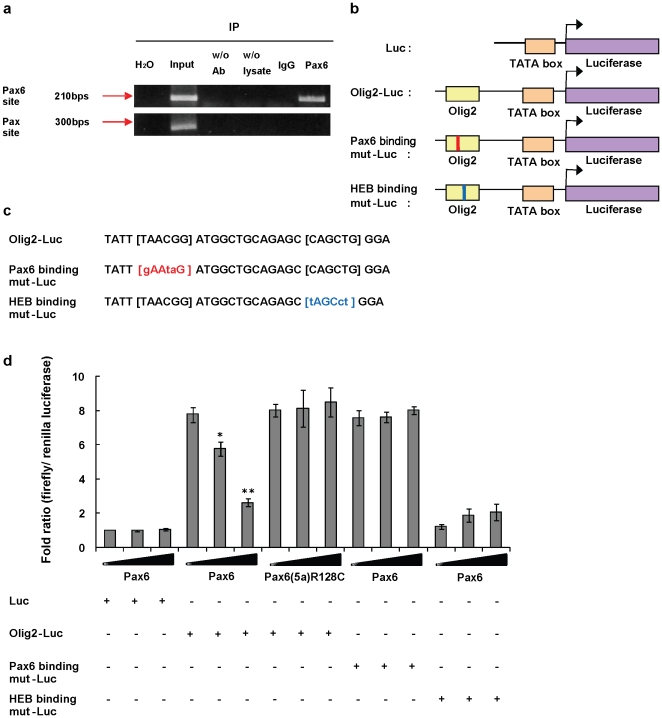
Pax6 represses *olig2* transcription by binding to the promoter region of the *olig2* gene. (**a**) Chromatin immunoprecipitation in P4 mouse forebrain lysate indicates that Pax6 binds to the promoter region of *olig2*. “Input” refers to 0.1%pre-immunoprecipitation input, “w/o Ab” is control without primary antibody, “w/o lysates” is control without lysate, “IgG” is immunoprecipitation with a control IgG, which serves as negative control. (**b**) Schematics of reporter gene vector. Normal Olig2 promoter region (Olig2-Luc) and Pax6 or HEB binding core sequences mutated promoter region (Pax6 or HEB Binding mut-Luc) are placed upstream of the firefly luciferase gene. **(c)** Mutated Pax6 or HEB binding site sequences. (**d**) Reporter assay reveals that Pax6 acts as a transcriptional repressor to *olig2*. Overexpression of *pax6* inhibits the *olig2* promoter reporter gene transcription, while the DNA binding site mutant *Pax6(5a) R128C* does not. A mutation in the Pax6 binding site abolishes the repression effect. Mutation in the HEB binding site abolishes luciferase activity, with or without Pax6. Each result represents a mean ± S.D. of three independent experiments carried out in triplicate. ^*^, by student's *t* test *p*<0.05,^ **^, *p*<0.01.

Based on the *in silico* findings, we predicted that Pax6 can associate with the *olig2* promoter. To confirm whether Pax6 binds to the *olig2*, we performed chromosomal immunoprecipitation (ChIP) experiments with lysates from P4 mouse forebrain (**Fig. 6a**). The same experiment with P2 rat SVZ gave the same result (not shown). By immunoprecipitation with a Pax6 antibody, followed by PCR with primers that flanked the putative Pax6 binding sequence in the *olig2* promoter, we amplified that specific Pax6 binding DNA sequence (**Fig. 6a**). A negative control and a control immunoprecipitation with IgG did not result in the amplified sequence. Furthermore, when we used a primer set that covers the region of the more generic Pax binding site, we did not find any amplified sequence.

### Pax6 represses *olig2* transcription and leads to lower Olig2 protein levels

To determine if Pax6 binding influenced transcriptional changes of *olig2*, we performed luciferase reporter assays. We made a firefly luciferase construct that contains almost 2.5 kb upstream of the *olig2* TSS (*Olig2-pLuc MCS*), including the putative Pax6 binding site (**Fig. 6b**), and co-transfected this construct with a *pax6* (*p3XFLAG-pax6*) or *eGFP* (*pEGFP*) (as control) expression construct into the oligodendrocyte precursor cell line, Oli-Neu. This line has high endogenous Olig2 levels but no detectable Pax6 (data not shown). The effects of Pax6 overexpression were determined in the presence of 0, 50, or 100 ng of the *pax6* expression construct by detecting luciferase activity after 48 hrs. We found that 100 ng of *pax6* construct led to over a 75%decrease in *Olig2-pLuc MCS* transcriptional activity, compared to basal *Olig2 pLuc* activity (p<0.01) (**Fig. 6d**). Overexpression of a Pax6 that does not bind DNA (binding site mutant construct, *Pax6(5a) R128C* [29], did not repress *olig2* promoter-luciferase activity (**Fig. 6d**).

To examine the Pax6 and HEB binding sequences in the *olig2* promoter further, we also generated two more *Olig2-pLuc* constructs. In one, the 3 most highly conserved base pairs of the Pax6 core-binding site were mutated and in the other, the 3 most highly conserved base pairs in the HEB site were mutated (**Fig. 6b, c**). Mutation of the Pax6 binding site resulted in luciferase activity as high as with the *Olig2-pLuc* vector in the absence of Pax6 (recall that the Oli-Neu cell line does not contain detectable Pax6) (**Fig. 6d**). Furthermore, expressing Pax6 did not diminish this activity (**Fig. 6d**). This result implies that the inhibition of *Olig2-pLuc* transcription that we observed in the presence of Pax6 (above) is a direct result of Pax6 binding to the Pax6 site.

Mutation of the HEB binding core sequence markedly decreased the baseline transcriptional activity of *olig2*, reducing it to that observed in the *pLuc* control (the *pLuc* vector without the *olig2* promoter) (**Fig. 6d**). This result indicates that the HEB site is required for the high basal *olig2* transcription in these cells. When we expressed Pax6 along with the HEB site mutant construct, we saw a small increase in luciferase activity over the low control level (**Fig. 6d**). This may suggest that Pax6 has a weak activator effect in the context of the mutated HEB site. The more important conclusion is that the Pax6 inhibition of *olig2* transcription requires an intact HEB binding site. We do not yet know the mechanism underlying this inhibition, but suggest possibilities below (see Discussion).

**Figure 7:**
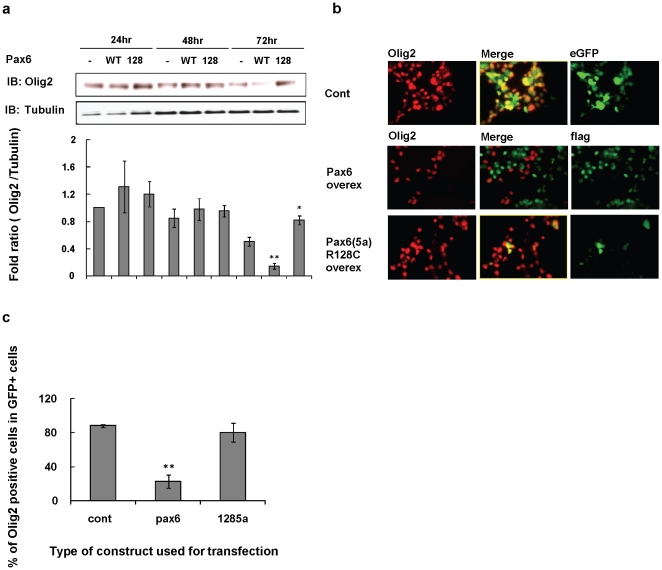
Overexpression of Pax6 decreases Olig2 protein levels. (**a**) Proteins for Western blotting were analyzed 24, 48, and 72 hrs after transfection. (**a**) Oli-Neu cells were transfected with flag-tagged WT Pax6 (WT), Pax6(5a)R128C (128), or eGFP control vector (-). (**b**) Immunostaining analysis 72 hrs after transfection shows that overexpressed Pax6 decreases Olig2 levels in Oli-Neu cells. **(c)** Histogram summarizing percentage of Olig2+ cells in Oli-Neu cells after transfection**.** Each result represents a mean ± S.D. of three independent experiments. At each time point (24, 48, 72 h) we compared the Olig2 protein band seen after transfection with the WT Pax6 vector and the mutant Pax6 vector with the Olig2 band seen after transfection with the control vector. ^*^ by student's *t* test *p*<0.05,^ **^ by student's *t* test *p*<0.01.

To determine if the transcriptional repression resulted in lower levels of the Olig2 protein, we examined the endogenous 32 kDa Olig2 protein levels in Oli-Neu cells after transfection of a flag-tagged *pax6* construct (*p3xFLAG-Pax6*) (**Fig. 7a**). Western blotting revealed that the Olig2 protein level was decreased more than 80%by the overexpression of *pax6* by 72 hrs after transfection (p<0.01). In contrast, Olig2 levels were not decreased after overexpression of a control *eGFP* construct or the Pax6 DNA binding site mutant, *Pax6(5a) R128C* construct (**Fig. 7a**). We also investigated Olig2 protein expression levels by immunofluorescence staining for Olig2 after transfection of flag-tagged *pax6* construct into Oli-Neu cells (**Fig. 7b**). Almost 77%of cells transfected with the *pax6* construct appeared Olig2- or weak Olig2+ (p<0.01), whereas cells transfected with a control GFP empty vector or the *Pax6(5a) R128C* vector sustained their endogenous Olig2 levels (**Fig. 7b, c**).

## Discussion

**Figure 8:**
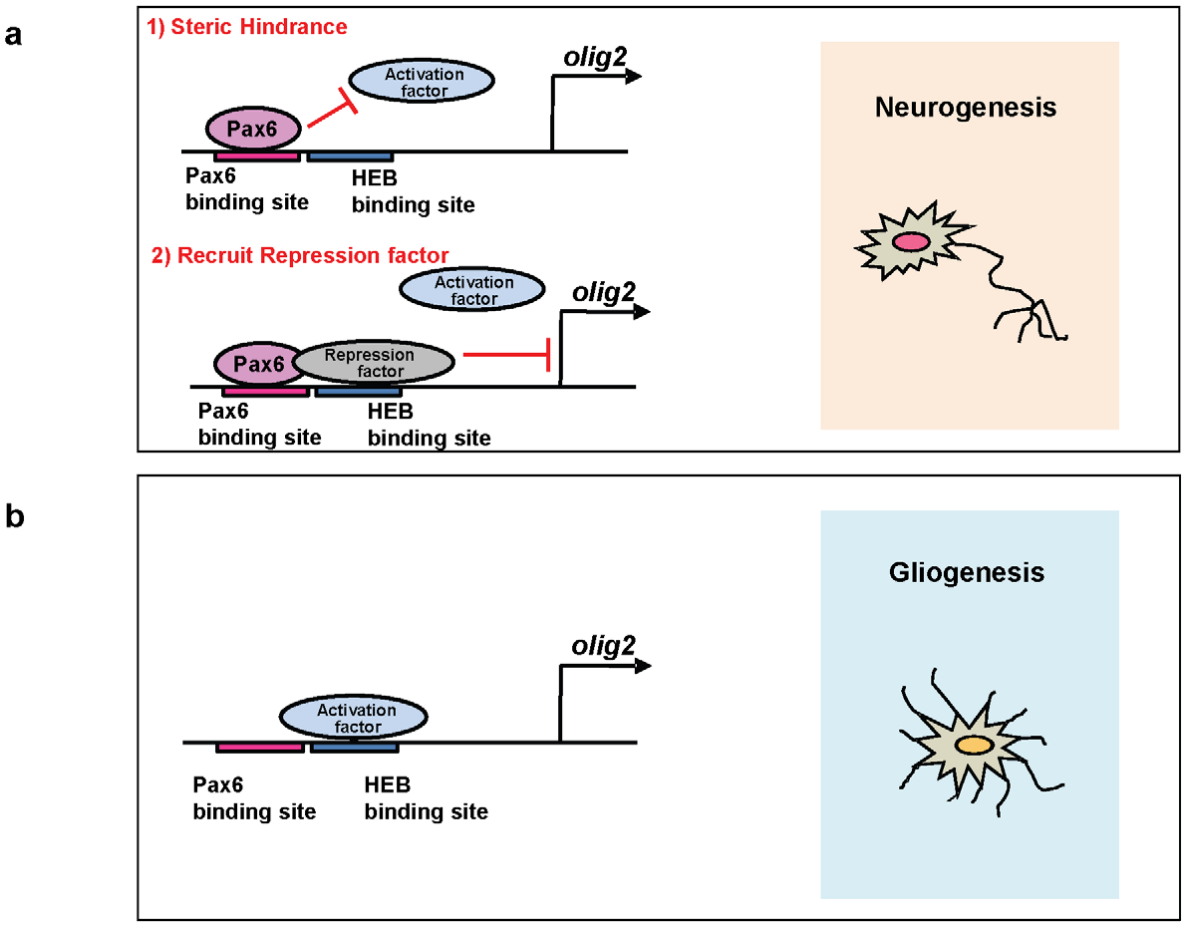
A model depicting the possible *olig2* regulation mechanism by Pax6. (**a**) In this model, Pax6 protein represses the *olig2* gene by binding on the *olig2* promoter region. This binding either could inhibit the binding to an activator to the HEB binding site or recruit a repressor to the HEB site. Either would inhibit *olig2* and promote neurogenesis. (**b**)Without Pax6, unknown activator complex is recruited and activates *olig2* transcription, leading to higher Olig2 levels and gliogenesis.

While Pax6 acts mostly as a transcriptional activator, there are several reports of transcriptional repressor activity, which is independent of the Pax6-transactivation domain [11]–[13]. In addition, Tang et al., [30] determined a modest intrinsic repressor activity of Pax6. In our study, we provide evidence for transcriptional repression of *olig2* transcription by Pax6. We found that a consensus Pax6 site and an HEB site on the *olig2* promoter are separated by only about a dozen base pairs. Our luciferase data strongly suggest that a putative activator complex binds to the HEB site and activates *olig2,* since a mutation in the HEB binding site decreases the basal *olig2* transcription to control levels. (**Fig. 6**) However, mutating the Pax6 binding site did not result in increased *olig2* transcriptional activity. Thus, it is possible that Pax6 represses *olig2* transcription by inhibiting the binding of a transcriptional-promoting complex at the HEB site or recruits a transcriptional-inhibiting complex instead (**Fig. 8**). Further study of this promoter site and a definition of the transcriptional complexes that bind to that site are in order. Here, we showed our results with using both mice and rats, those results suggest that Pax6 and Olig2 expression and function in both species are similar at the same developmental time stage.

The majority of Ki67+ perinatal SVZ cells that we think of as neurogenic and/or gliogenic progenitor cells either express Pax6 or Olig2 (**Fig. S4**). Olig2 expression has been reported in a subset of ‘type B' cells and in transit –amplifying (‘type C') cells in the adult mouse SVZ, and these cells have gliogenic potential [25], [31]. Some of the type C cells in the adult SVZ express Pax6 and generate olfactory interneurons in the adult SVZ [32]. It is not clear if B, C, and A cells can be clearly defined in the neonatal SVZ, but the large cycling population is likely to be most similar to a mix of C and A cells.

We don't know yet if the Pax6+ and Olig2+ positive cells in the neonatal SVZ are equivalent to C cells in the adult SVZ. In the perinatal SVZ, most of the Pax6 cells expressed an early neuroblast marker, TuJ1, whereas the Olig2+ cells were TuJ1- and instead predominantly expressed NG2 and ZebrinII, suggesting that they are glial precursors (28). We also find radial glial cells, defined by a long, radial morphology and the expression of RC2 to be Pax6+ or Olig2+ [20] but not both. We cannot at this time conclude that the Pax6+ radial glia (or progenitor cells) are neurogenic and the Olig2+ radial glia (or progenitor cells) are gliogenic in this perinatal time, but our findings are consistent with previous data supporting Pax6 as an intrinsic neuronal fate determinant [16], and Olig2 as a glial fate determinant [20].

Our observations in this and in a previous study [20] indicate that neonatal SVZ cells are developmentally plastic enough to change their Pax6 or Olig2 expression levels after overexpression of the other factor and that this change appears to be sufficient to promote the early acquisition of neuronal or glial fates, respectively, and change the migration patterns appropriately. Cell fate and migration patterns are strongly linked for SVZ cells, since immature glia migrate radially into overlying white matter, cortex, and striatum, whereas immature neurons migrate into and along the RMS. However, some cortical GABAergic interneurons are generated from SVZ cells, particularly in the frontal cortex [28], although it was not reported whether these cortical interneurons expressed *pax6.* Glial precursor migration appears to occur along radial glial processes and axons [34], [35], whereas neuronal precursor migration takes place in a “chain migration” in which neuroblasts migrate over each other [3]. This coupling of fate and migratory patterns may be determined by Pax6 and Olig2, but we do not yet know if there are specific adhesion molecule or migration genes that are regulated by these transcription factors. Studies in non-CNS development have identified several adhesion molecule genes regulated by Pax6 [36], but it is not known if any of these plays a role in specifying glial vs. neuronal migration. The analysis of genes downstream of *pax6* and *olig2* may shed light on the molecular coordination between fate determination and migration patterns.

In the adult CNS there is evidence for neuronal-glial fate plasticity. Recently, Jablonska et al.(36) showed that lysolecithin-induced demyelination in the white matter overlying the adult SVZ caused Pax6+/Glutamic acid decarboxylase 65+/Doublecortin+ precursor cells in the adult SVZ to switch their fates from olfactory interneurons to oligodendrocytes and to migrate into the overlying corpus callosum. We do not know if the cells require Pax6 loss and Olig2 expression to switch fates, but if so, a switch implies neuronal-glial plasticity of Pax6+ cells and is consistent with our observations in the neonatal SVZ.

What might be the function of this cross-repression in the neonatal SVZ? This is an area of high cell density and active cell migration that generates both neurons and glia. In addition, the neonatal forebrain SVZ shows the expression of factors that regulate neuronal and glial fate decisions, such as Shh and BMP (*in situ* hybridization data, S. Ivkovic personal communication). However, it appears unlikely that *olig2* and *pax6* actually regulate the initial fate determination of SVZ cells. Indeed, SVZ cells may originate from precursors already expressing *pax6* or *olig2*. The migratory cell population of the neonatal SVZ cells has at least two sources: 1. Precursors generated ventrally in embryonic times that migrate into the dorso-lateral SVZ [26] and 2. radial cells of the dorsal telencephalon, which generate neurons and glia in the early postnatal brain [38]. We have found Pax6+ and Olig2+ cells in this dorsal radial cell population, defined by RC2 expression and morphology, and there is no overlap between the expression of the two proteins in the RC2+ population ( [20] and this paper). Thus, it is possible that a glial vs. neuronal fate determination takes place at an earlier, radial progenitor stage. We would have to trace the fates of the Pax6+ and Olig2+ radial populations to confirm this idea. However, since the expression of *pax6* and *olig2* is sufficient to force glial precursors to begin to differentiate along a neuronal developmental pathway, and force neuronal precursors to begin to differentiate along a gliogenic pathway, respectively, these two transcription factors must be situated at critical positions in a transcriptional network that underlies the development of interneuron and glial phenotypes.

How specific is *olig2* expression for a committed glial lineage in the neonatal SVZ? Could *olig2* be expressed during an early stage of neuroblast development? For example, could *olig2* be expressed briefly in transit-amplifying cells that will eventually differentiate into Pax6+ neuroblasts? Our observations with the *floxed eGFP-stop-Ds-red* expressing lentivirus in the *olig2*-*Cre* mouse brains suggest that this is not the case. If an Olig2+ stage were an obligatory step in the early development of neuroblasts, then we would have expected to see Ds-red+/Pax6+ cells in the RMS. Such cells were very rare, however, and it will require a much more extensive analysis to characterize them. Indeed, the vast majority of Ds-red+ cells were also Olig2+ and had migrated radially like glioblasts.

In thinking about the function of Pax6 and Olig2 cross-repression, one needs to consider that, despite the apparent committed fates of glioblasts and neuroblasts in the normal neonatal SVZ *in vivo*, these cells still retain developmental plasticity. For example, neonatal SVZ cells labeled by retrovirus *in vivo* can generate clones containing both neurons and glia when removed from the SVZ and grown in culture [6]. Adult SVZ cells that are Pax6+ can become glia after white matter demyelination [37]. Once an early neuronal vs. glial fate decision has begun, this cross-repressive feedback system would serve to stabilize a cell's fate and inhibit other fates. This would prevent the development of immature cells with mixed neuronal and glial characteristics, for example. Thus, Pax6 and Olig2 could form a reciprocal-repression feedback loop to keep an immature cell firmly headed along neuronal or glial differentiation once an initial lineage progression has begun.

## Materials and Methods

### Animals

Olig2-GFP and Olig2-Cre mice (B.G. Novitch and T.M. Jessell, unpublished observation) were generated by disruption of the coding region of Olig2 with an IRES-eGFP and IRES-Cre cassette. BAC clone PAX6-GFP mouse was a gift from Prof. David Price, Univ. of Edinburgh. We also used Sprague Dawley rats for retrovirus injection. All animal work was performed according to Institutional Animal Care and Use Committee guidelines of Columbia University. Mouse protocols #AAAA4951, AAAA5839; rat protocols AAAA4417, AAAA8919 were approved by the Institutional Animal Care and Use Committee of Columbia University.

### Immunohistochemistry

All tissue sections and antibodies are prepared as described previously (Marshall et al., 2005). Primary antibodies were rabbit anti-Pax6 (Chemicon, 1∶1000), goat anti-Pax6 (Sigma, 1∶1000), rabbit anti-Olig2 (Chemicon, 1∶1000), guineapig anti-Olig2 (1∶20,000, gift of David Rowitch, UCSF), rabbit anti-Olig2 (ABCAM, 1∶1000), mouse anti-ß-tubulin (TuJ1) (Covance, 1∶200), mouse anti-RC2 antibody (Developmental Studies Hybridoma Bank, 1∶5), rabbit anti-Ki67 (ABCAM, 1∶500), mouse anti-flag (Sigma, 1∶200), rabbit anti-flag (Sigma, 1∶100), and mouse anti-ds-red (BD Biosciences, 1∶1000).

### Microscopy

Histological sections and cell cultures were examined and photographed using a Zeiss Axiophot 200 fluorescence microscope equipped with an Axiocam (Zeiss, Thornwood, NY) and Openlab imaging software (Improvision, Lexington, MA). Micrographs were further processed into composite figures using Adobe Photoshop.

### *In silico* approach

The sequence that spanned 10,000 bps upstream to 200 bp downstream of the *olig2* or *pax6* gene's transcriptional start site (TSS) was extracted from the University of California at Santa Cruz Genome Bioinformatics website (http://genome.ucsc.edu). TRANSFAC® Professional 8.3 package (http://www.biobase.de/) was used for the computational identification of transcription factor binding sites. In the matrix search for transcription factor binding sites, only high quality matrices are applied to the MATCH^ TM^ 2.2 program to minimize false positives. A phylogenetic footprinting method that is based on the fact that biologically important regulatory elements have been selectively conserved during the evolution was applied to the results from the matrix search. In this analysis, ClustalW was used for multiple alignments between the *olig2* promoter sequences from multiple species.

### Chromatin immunoprecipitation assay (ChIP)

The Millipore ChIP assay protocol was used for P2 rat or P4 mouse forebrain cells using the rabbit anti-Pax6 antibody, or a control normal rabbit IgG (Santa Cruz). PCR amplification was performed using following primer sets.

*olig2* promoter Pax6 binding site: Fwd 5′CAAGAGTGGTCCTCACATGC3′

Rev 5′CCTTCTGGGGCATTGTTTT 3′

*olig2* promoter Pax binding site: Fwd 5′AATAGGGAACCAGGGAATCG 3′

Rev 5′CTCCTTCAACCAGGAAGACG3′

### Construction of reporter plasmids, and luciferase reporter assay

*olig2* promoter Luciferase plasmid was constructed by inserting upstream regions of *olig2* into pLuc-MCS (Stratagene).

*Bam*HI Fwd 5′CGGGATCCGAATCGCTGCCTGAATGCTA3′

*Sal*I Rev 5′ACGCGTCGACGATGCTGCGGTGAGTGTAGA3′

The *olig2* promoter insert was amplified by PCR using primers, digested with *Bam*HI, and *Sal*I, inserted into the respective site of pLuc-MCS (Stratagene). *Olig2* promoter luciferase constructs with a 3 bp point mutation either in the Pax6 or the HEB binding sequence were generated using QuickChange® Site Directed Mutagenesis Kit (Stratagene) and the following primers, shown with the mutated 6 bp indicated in bold:

Pax6 mut luciferase

Fwd 5′CATGTTTTAAATACATATT[**G**AA**TA**G]ATGGCTGCAGAGCCAGCTGGG3′

Rev 5′CCCAGCTGGCTCTGCAGCCATCTATTCAATATGTATTTAAAACATC3′

HEB mut luciferase

Fwd 5′CACGGATGGCTGCAGAGC[**T**AGC**CT**]GGAAACACGCGGGTCGG3′

Rev 5′CCGACCCGCGTGTTTCCAGGCTAGCTCTGCAGCCATCCGTG3′

For the luciferase assay in the oligodendrocyte precursor cell line Oli-Neu (gift of Dr. Jacqueline Trotter, Univ. of Mainz), 50 ng of *olig2* promoter reporter plasmid DNA was co-transfected into 2×10^4^ cells with 0, 50, and 100 ng of *pax6* expressing p3XFLAG-Pax6 (gift from Dr. Cvekl, Albert Einstein College of Medicine), 10 ng of *Renilla* luciferase expressing pRL-TK (Promega), and pEGFP (Clontech) for adjusting transfected DNA amount into 150 ng using Fugene (Roche). We used a DNA binding paired domain mutant construct, Pax6(5a)R128C (from Dr. Cvekl) instead of using the Pax6 expression vector.

### Western Blot Analysis

Cell lysates were prepared in RIPA buffer (Sigma) 24, 48, and 72 h after transfection. Equal amounts of lysates (15 µg protein) were resolved on a NuPAGE Novex 10%Bis-Tris gel (Invitrogen). Primary antibodies used were: rabbit anti-Olig2 (ABCAM, 1∶1000), rabbit anti-Pax6 (Chemicon, 1∶1000), mouse anti-ß-Tubulin (Santa Cruz, 1∶1000). After stripping the Olig2 or Pax6 antibodies, gels were re-probed with anti-α–tubulin for normalization.

### Viral vectors and virus preparation

For Pax6 retrovirus, all fragments encoding Pax6 or mutant Pax6 cDNAs were subcloned into the pQCXIX (Clontech). Coding fragments were as follows: *m-pax6* (*Bam*H1 *and Hind*III fragment from *p3xflag-Pax6*), *m-pax6(5a) R128C* (*Bam*H1 *and Hind*III fragment from *p3xflag-Pax6(5a) R128C*). The *pax6-R128C* encodes a mutant protein that does not bind DNA (30).

To generate lentivirus expressing control *eGFPstop-loxp-IRES-dsred* lenti virus, we first inserted the coding sequence (*Nhe*I and *Not*I fragment from *p212 pCMV-EGFP/RFP* (addgene)) into the pLion (addgene). To generate *loxp-eGFPstop-loxp-IRES-flag-pax6*, inserts were amplified from *p3xflag Pax6* by genomic PCR.

*Sac*II Fwd 5′AAACCGCGGACCACCATGGACTACAAAGACCATGACG3′

*Not*I Rev 5′AAAGCGGCCGCTTACTGTAATCGAGGCCAGTAC3′

We cleaved the control lentivirus with *Nhe*I and *Not*I and ligated the flag-tagged Pax6 insert (*Sac*II and *Not*I fragment). Viral stocks were concentrated by centrifugation and titered to 1×10^6^ cfu/ml using 3T3 cells 48 h post-transfection.

Retrovirus generation was described previously [20]. For lentivirus, lentiviral vectors were cotransfected with pCMV-dR8.2 dvpr and *pCMV-VSVG* into HEK-293T (Clontech) cells using Lipofectamine 2000 (Invitrogen). Viral stocks were concentrated by centrifugation and titered to 1–3×10^5^ cfu/ml using 293 cells 48 h post-transfection.

### Viral injections

Retroviral injections into neonatal rat pups were done as previously described [20]. Lentiviral injections were performed on P3 *olig2*-Cre mice. After anesthetization of P3 mouse by hypothermia, 0.1 µl of lentivirus suspension was injected unilaterally at a rate of 0.02 µl/min using a 10 µl Hamilton (Reno, NV) syringe. Coordinates used for mouse pups was 0 mm bregma line, 0.8 mm lateral, and 1.5 mm depth.

### Mouse brain cell dispersion and Flow Cytometry

Detailed methods are described in the Legend to Figure 4.

### Statistical analysis

Statistical analysis was done with the unpaired Student's *t*-test; *n* corresponds to the number of all cells, brains or data analyzed. For the injections, the S.E.M. represents the variance between different injections into different animals; that is, a single data point represents all of the fluorescence-positive cells counted in one animal.

## Supporting Information

Figure S1 **Pax6 and Olig2 express exclusively in the postnatal p2 rat brain** **SVZ region.** Pax6 and Olig2 show exclusive expression pattern in neonatal SVZ, as indicated by immunofluorescence staining of P2 rat brain. (**a**–**c**) Micrograph of Olig2+ (**a**), Pax6+ (**c**) or merged (**b**) cells at coronal plane of neonatal P2 rat SVZ. White box depicts diagram of coronal plane neonatal SVZ. Red inset represents the site of picture taken on the diagram of coronal brain plane. Pax6 immunostaining in green, Olig2 in red. SVZ; subventricular zone, LV; lateral ventricle, WM; white matter. Scale bars, 50 µm.
(TIF)

Figure S2 **Cell marker expression of Olig2+ and Pax6+ SVZ cells.** Pax6 or Olig2 expression in Zebrin II+ (**a**, **b**), RC2+ (**d**, **e**), TuJ1+ (**g**, **h**), or NG2+ (**j**, **k**) SVZ cells, as indicated by immunostaining of P4 mouse parasagittal sections. (**c**, **f**, **i**, **l**) Histogram summarizing percentage of marker positive cells (Zebrin II + in (**c**), RC2+ in (**f**), TuJ1+ in (**i**), and NG2+ in (**l**)) in either Pax6 (left lane) or Olig2 (right lane) positive populations in P4 mouse postnatal SVZ. Inset (**m**) depicts the area of photomicrographs shown. SVZ; subventricular zone, LV; lateral ventricle, WM; white matter. Scale bars 50 µm.
(TIF)

Figure S3 **Control retrovirus infected cells exhibit a normal (mixed glial and neuronal) migration pattern.** Retrovirus was injected into the SVZ of P2 rats (n = 7) and analyzed 4d later. (**a**) Schematics of control retroviral constructs. (**b**, **c**) *pq-IRES-eGFP* infected cells migrate radially migrate into WM or remain in the SVZ (**b**) or tangentially into the RMS (**c**). (**d**) Diagrammatic distribution of infected cells and injection site. (**e**) Histogram analysis of the effects of control retrovirus infection on migratory behavior, presented as the percentage of infected cells per brain located in each area (mean ± SEM). (**f**, **g**) Schematics of *pax6* (**f**), and *pax6(5a) R128C* (**g**) retroviral constructs. The numbers of infected cells per brain ranged from 40 to 67. All data made with 4 dpi sections. Scale bars, 50 µm.
(TIF)

Figure S4 **Pax6 or Olig2 protein expresses within proliferating progenitor cells in postnatal SVZ.** (**a**, **b**) Proliferating cells in the parasagittal sections of the P4 mouse brain SVZ were detected using an antibody against proliferating cell marker Ki67. (**a**) Pax6+ cells were observed within proliferating (Ki67 positive; green) SVZ region. Pax6 immunostaining in red, Ki67 in green. (**b**) Olig2+/Ki67+ cells were detected within the SVZ region. Olig2 immunostaining in red, Ki67 in green. SVZ; subventricular zone, LV; lateral ventricle WM; white matter. Scale bars, 50 µm.
(TIF)

Figure S5 **Lentiviral derived flag is expressed exclusively by SVZ-derived** **neuronal lineage cells.** (**a–c**) Ds-red + cells (**a**) were overlapped with Olig2 (**c**) in SVZ. Panel (**b**) shows merged cells at 4 dpi. White arrows point Ds-red+/Olig2+ cells. Olig2 immunostaining in Cy5, Ds-red in red. (**d**) In RMS, flag positive cells were merged with neuronal marker TuJ1 4 dpi after stereotactic injection of Pax6 lentivirus into P2 mouse SVZ. TuJ1 immunostaining in Cy5, flag in red. SVZ; subvenrticular zone, LV; lateral ventricle, RMS; rostral migratory stream. Scale bars, 50 µm.
(TIF)

Figure S6 **Virus injected brains did not show any distinct cell death.** (**a**, **b**) Cell death of retrovirus injected brains was determined by immunofluorescence staining with a cleaved caspase 3 antibody (cCas3). eGFP immunostaining in green, cCas3 in red. Micrograph of eGFP and cCas3 immunoreactive cells in a parasagittal plane of postnatal rat SVZ injected with retro *pax6-eGFP* (**a**) or *pax6(5a) R128C-eG*FP virus (**b**). (**c**, **d**) Micrograph of either eGFP (**c**) or Ds-red (**d**) and cCas3 immunoreactive cells at parasagittal plane of control lentivirus injected postnatal mouse SVZ. eGFP immunostaining in green, Dsred in red, and cCas3 in Cy5. (**e**, **f**) Micrograph of either eGFP (**e**) or flag (**e**) and cCas3 immunoreactive cells at parasagittal plane of Pax6-lenti virus injected postnatal mouse SVZ. eGFP immunostaining in green, flag in red, and cCas3 in Cy5. SVZ; subventricular zone, LV; lateral ventricle, WM; white matter. Scale bars, 50 µm.
(TIF)
