# Supplementary figures and images for: Pax6 Expression Is Sufficient to Induce a Neurogenic Fate in Glial Progenitors of the Neonatal Subventricular Zone
Source: PLoS One. 2011 Jun 17;6(6):e20894. doi: 10.1371/journal.pone.0020894 (PMC3117849; doi:10.1371/journal.pone.0020894)

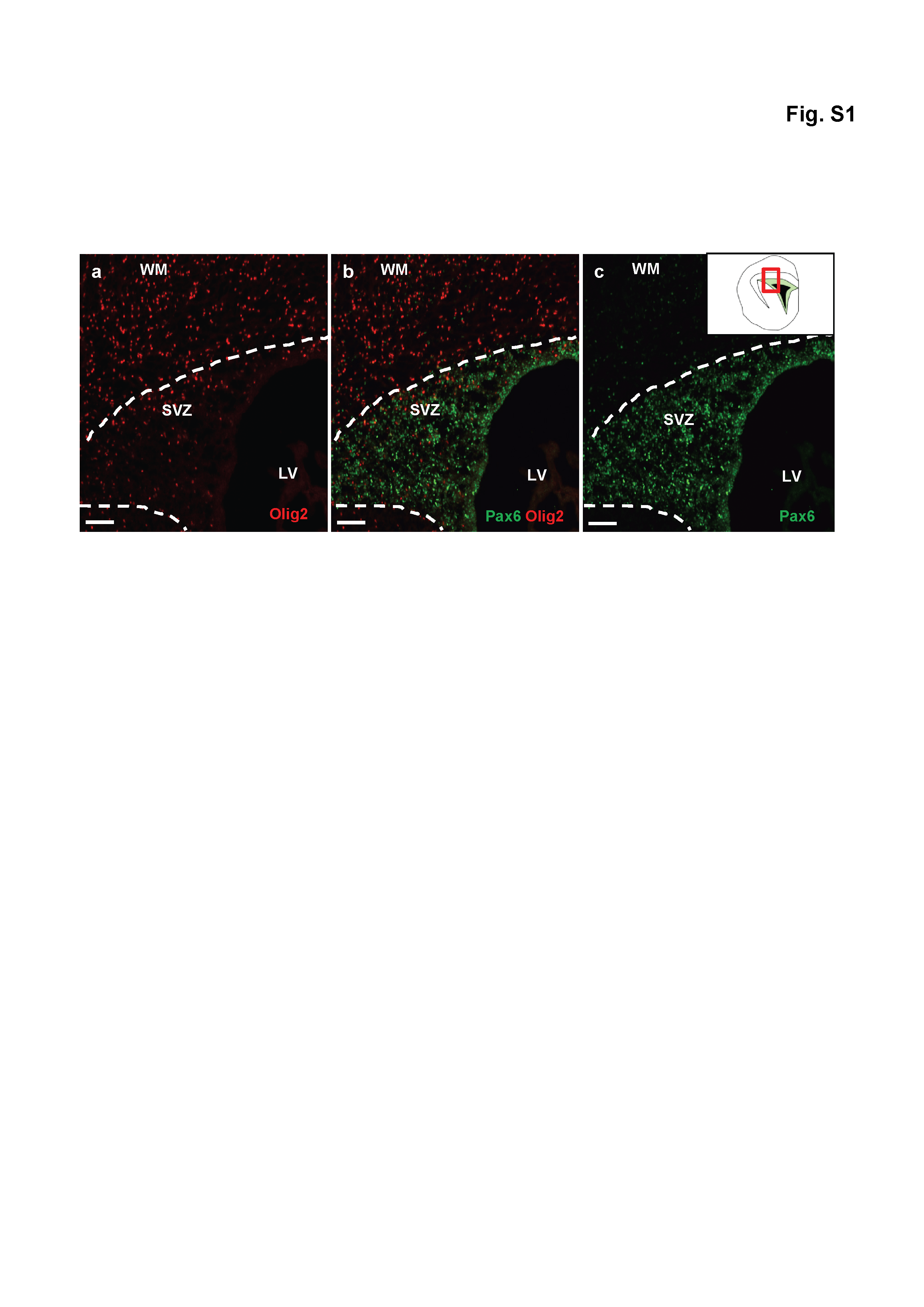

Supplement: Figure S1 — Pax6 and Olig2 express exclusively in the postnatal p2 rat brain SVZ region. Pax6 and Olig2 show exclusive expression pattern in neonatal SVZ, as indicated by immunofluorescence staining of P2 rat brain. (a–c) Micrograph of Olig2+ (a), Pax6+ (c) or merged (b) cells at coronal plane of neonatal P2 rat SVZ. White box depicts diagram of coronal plane neonatal SVZ. Red inset represents the site of picture taken on the diagram of coronal brain plane. Pax6 immunostaining in green, Olig2 in red. SVZ; subventricular zone, LV; lateral ventricle, WM; white matter. Scale bars, 50 µm. (TIF) [file pone.0020894.s001.tif]

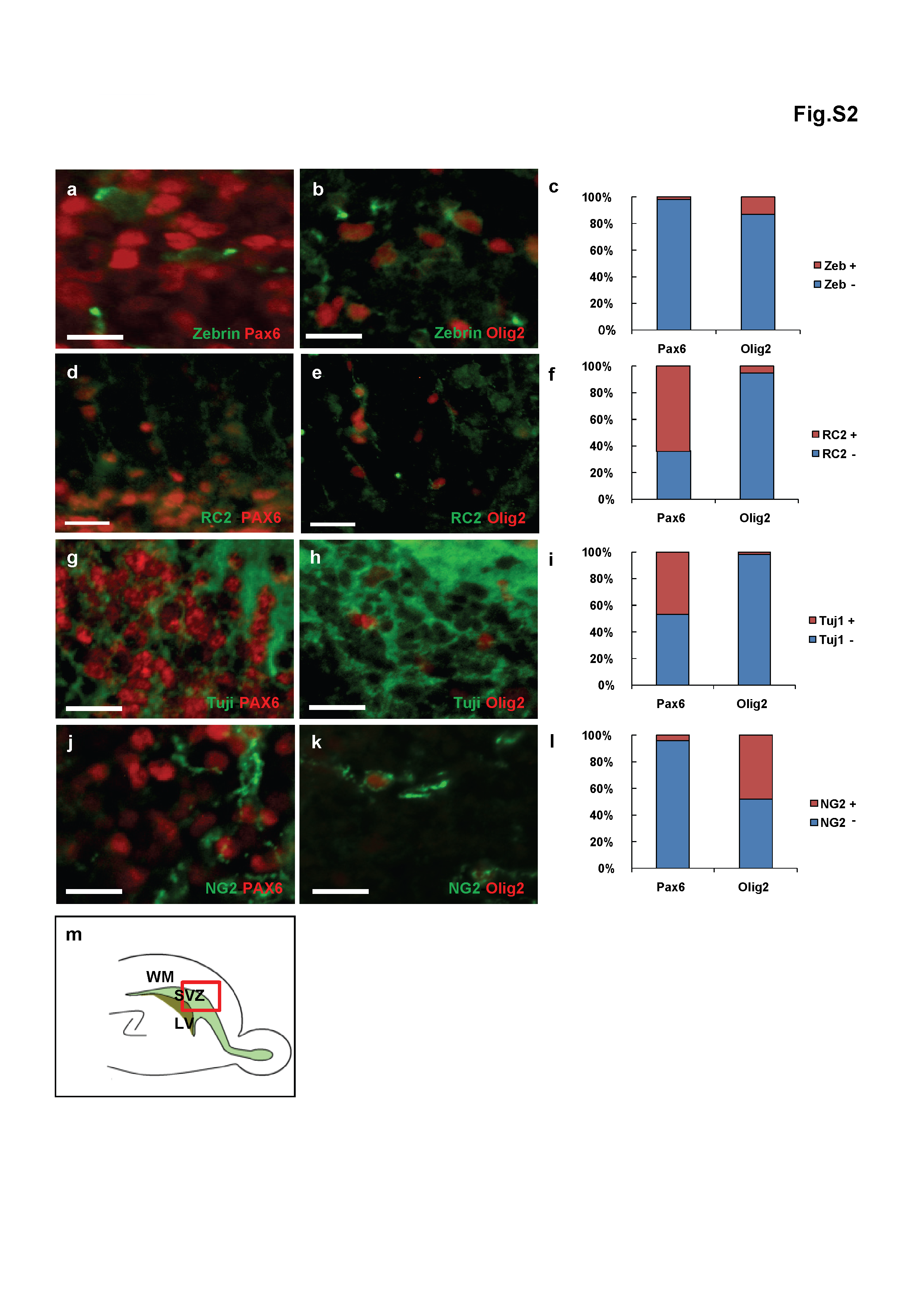

Supplement: Figure S2 — Cell marker expression of Olig2+ and Pax6+ SVZ cells. Pax6 or Olig2 expression in Zebrin II+ (a, b), RC2+ (d, e), TuJ1+ (g, h), or NG2+ (j, k) SVZ cells, as indicated by immunostaining of P4 mouse parasagittal sections. (c, f, i, l) Histogram summarizing percentage of marker positive cells (Zebrin II + in (c), RC2+ in (f), TuJ1+ in (i), and NG2+ in (l)) in either Pax6 (left lane) or Olig2 (right lane) positive populations in P4 mouse postnatal SVZ. Inset (m) depicts the area of photomicrographs shown. SVZ; subventricular zone, LV; lateral ventricle, WM; white matter. Scale bars 50 µm. (TIF) [file pone.0020894.s002.tif]

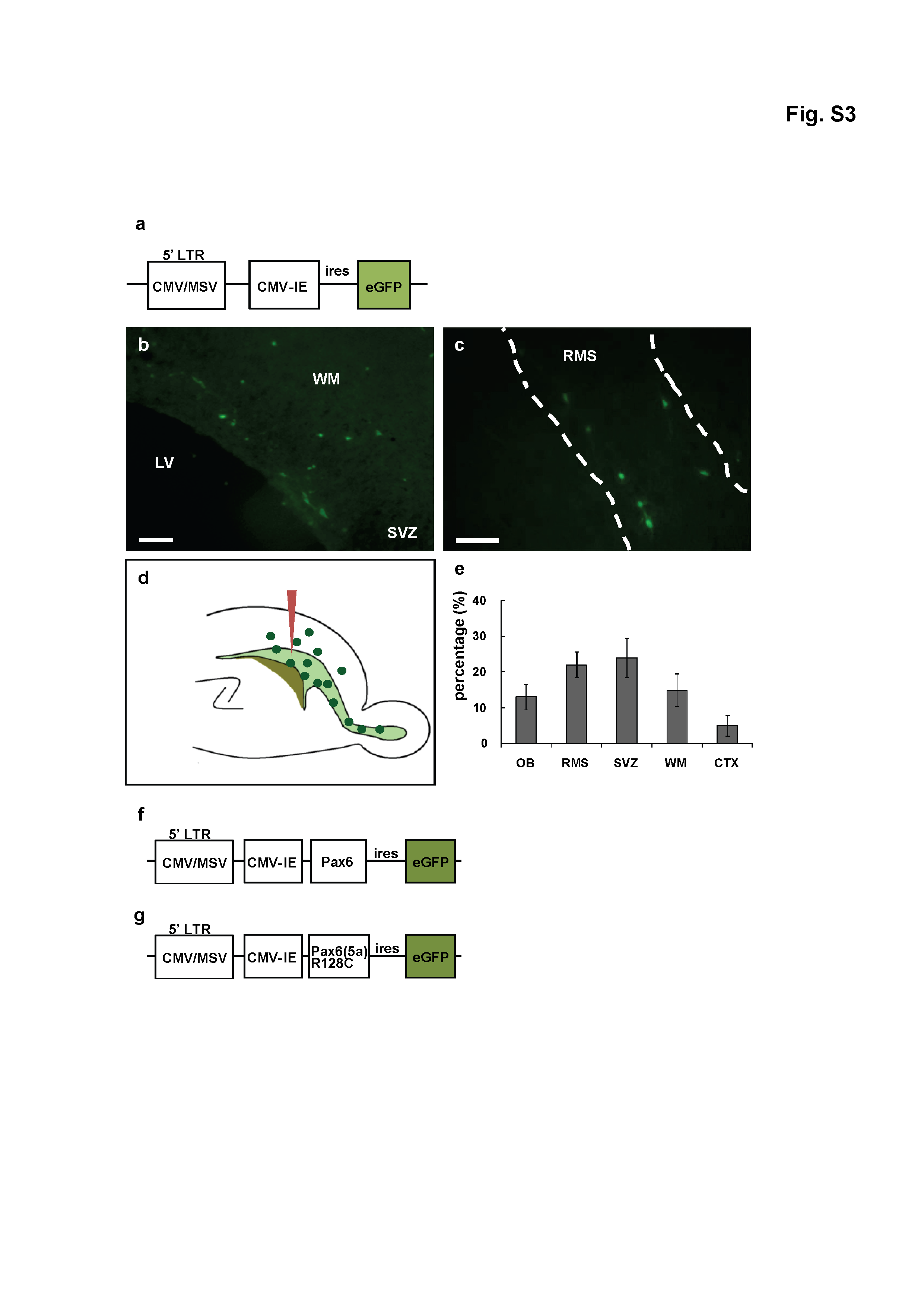

Supplement: Figure S3 — Control retrovirus infected cells exhibit a normal (mixed glial and neuronal) migration pattern. Retrovirus was injected into the SVZ of P2 rats (n = 7) and analyzed 4d later. (a) Schematics of control retroviral constructs. (b, c) pq-IRES-eGFP infected cells migrate radially migrate into WM or remain in the SVZ (b) or tangentially into the RMS (c). (d) Diagrammatic distribution of infected cells and injection site. (e) Histogram analysis of the effects of control retrovirus infection on migratory behavior, presented as the percentage of infected cells per brain located in each area (mean ± SEM). (f, g) Schematics of pax6 (f), and pax6(5a) R128C (g) retroviral constructs. The numbers of infected cells per brain ranged from 40 to 67. All data made with 4 dpi sections. Scale bars, 50 µm. (TIF) [file pone.0020894.s003.tif]

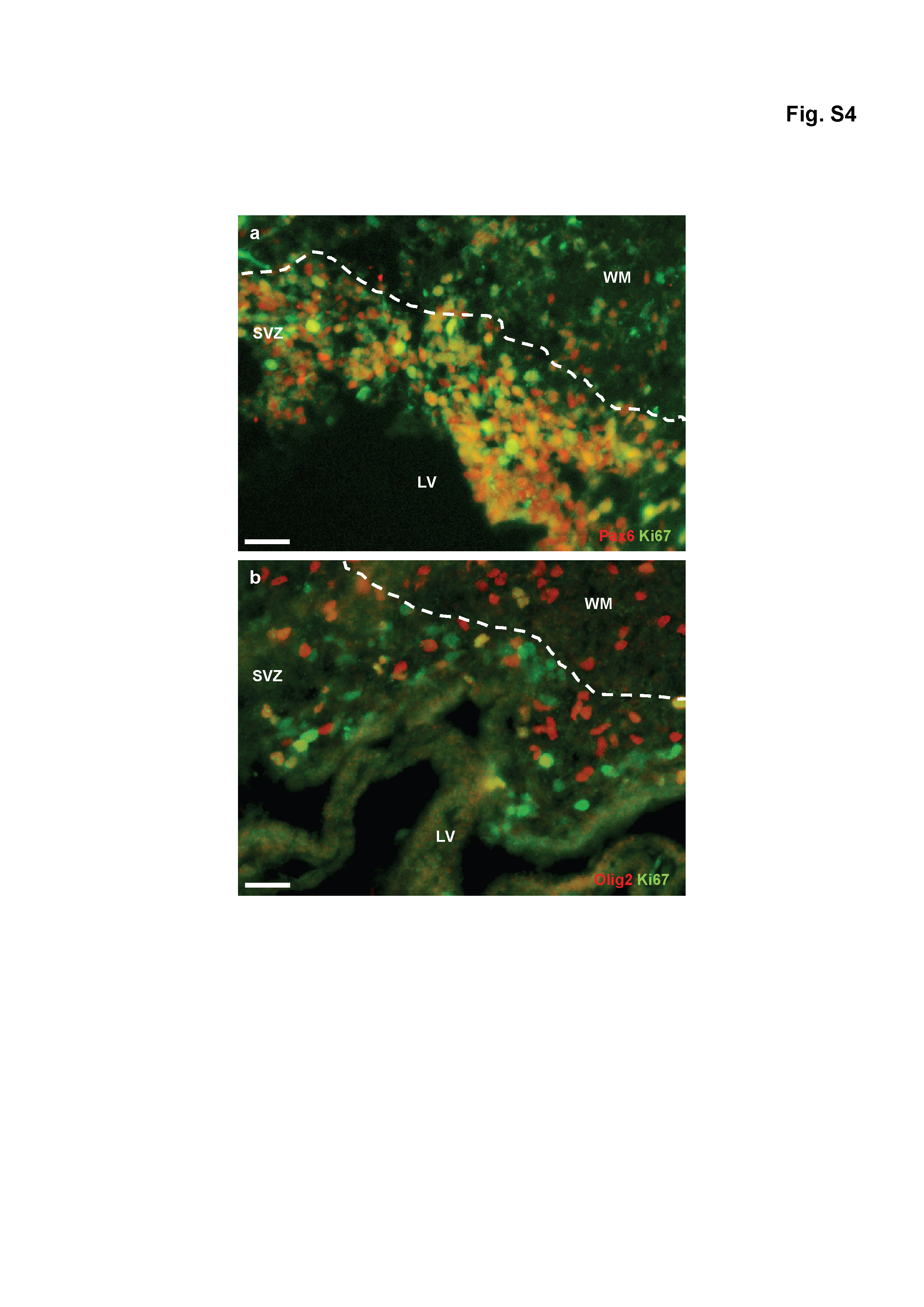

Supplement: Figure S4 — Pax6 or Olig2 protein expresses within proliferating progenitor cells in postnatal SVZ. (a, b) Proliferating cells in the parasagittal sections of the P4 mouse brain SVZ were detected using an antibody against proliferating cell marker Ki67. (a) Pax6+ cells were observed within proliferating (Ki67 positive; green) SVZ region. Pax6 immunostaining in red, Ki67 in green. (b) Olig2+/Ki67+ cells were detected within the SVZ region. Olig2 immunostaining in red, Ki67 in green. SVZ; subventricular zone, LV; lateral ventricle WM; white matter. Scale bars, 50 µm. (TIF) [file pone.0020894.s004.tif]

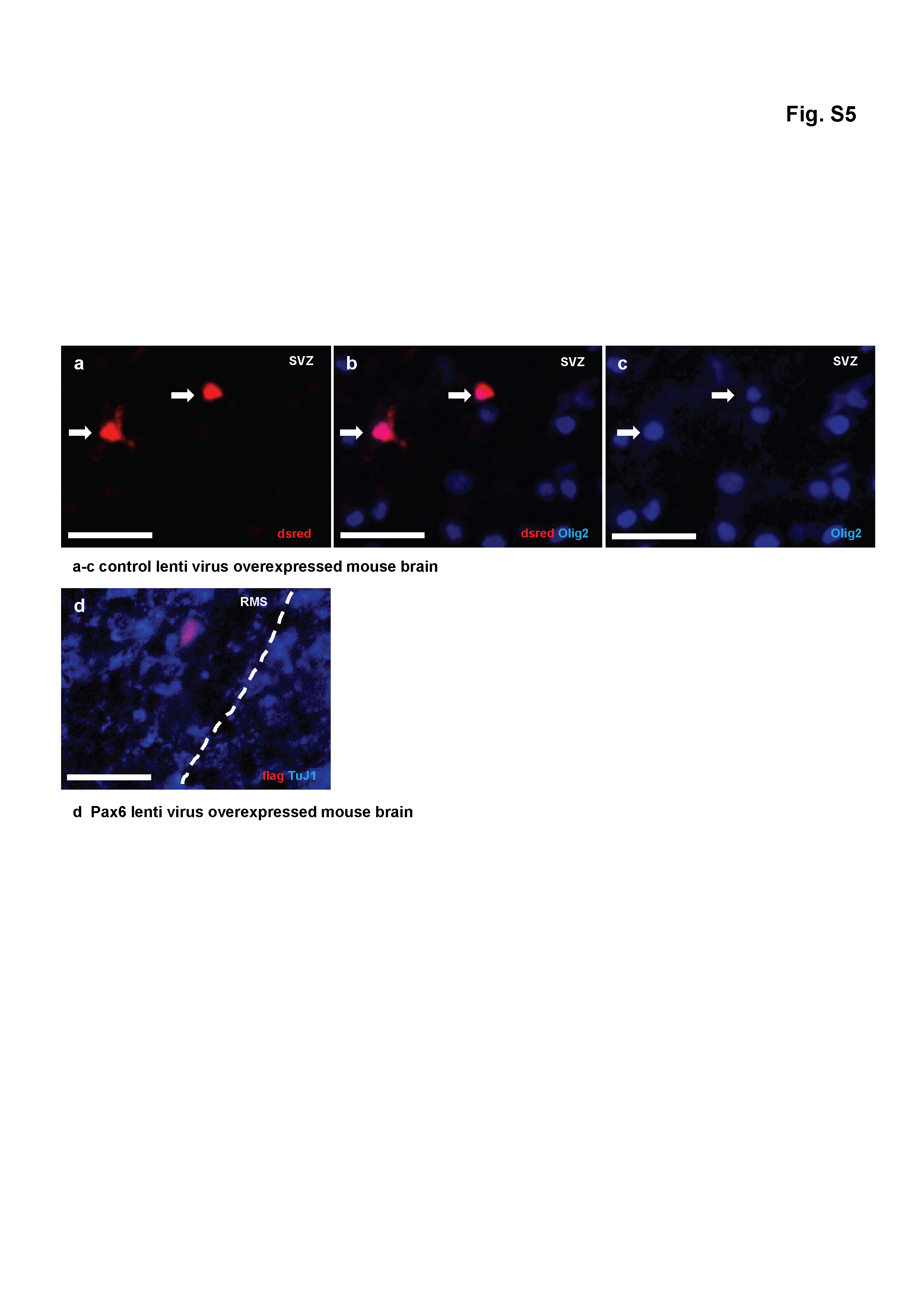

Supplement: Figure S5 — Lentiviral derived flag is expressed exclusively by SVZ-derived neuronal lineage cells. (a–c) Ds-red + cells (a) were overlapped with Olig2 (c) in SVZ. Panel (b) shows merged cells at 4 dpi. White arrows point Ds-red+/Olig2+ cells. Olig2 immunostaining in Cy5, Ds-red in red. (d) In RMS, flag positive cells were merged with neuronal marker TuJ1 4 dpi after stereotactic injection of Pax6 lentivirus into P2 mouse SVZ. TuJ1 immunostaining in Cy5, flag in red. SVZ; subvenrticular zone, LV; lateral ventricle, RMS; rostral migratory stream. Scale bars, 50 µm. (TIF) [file pone.0020894.s005.tif]

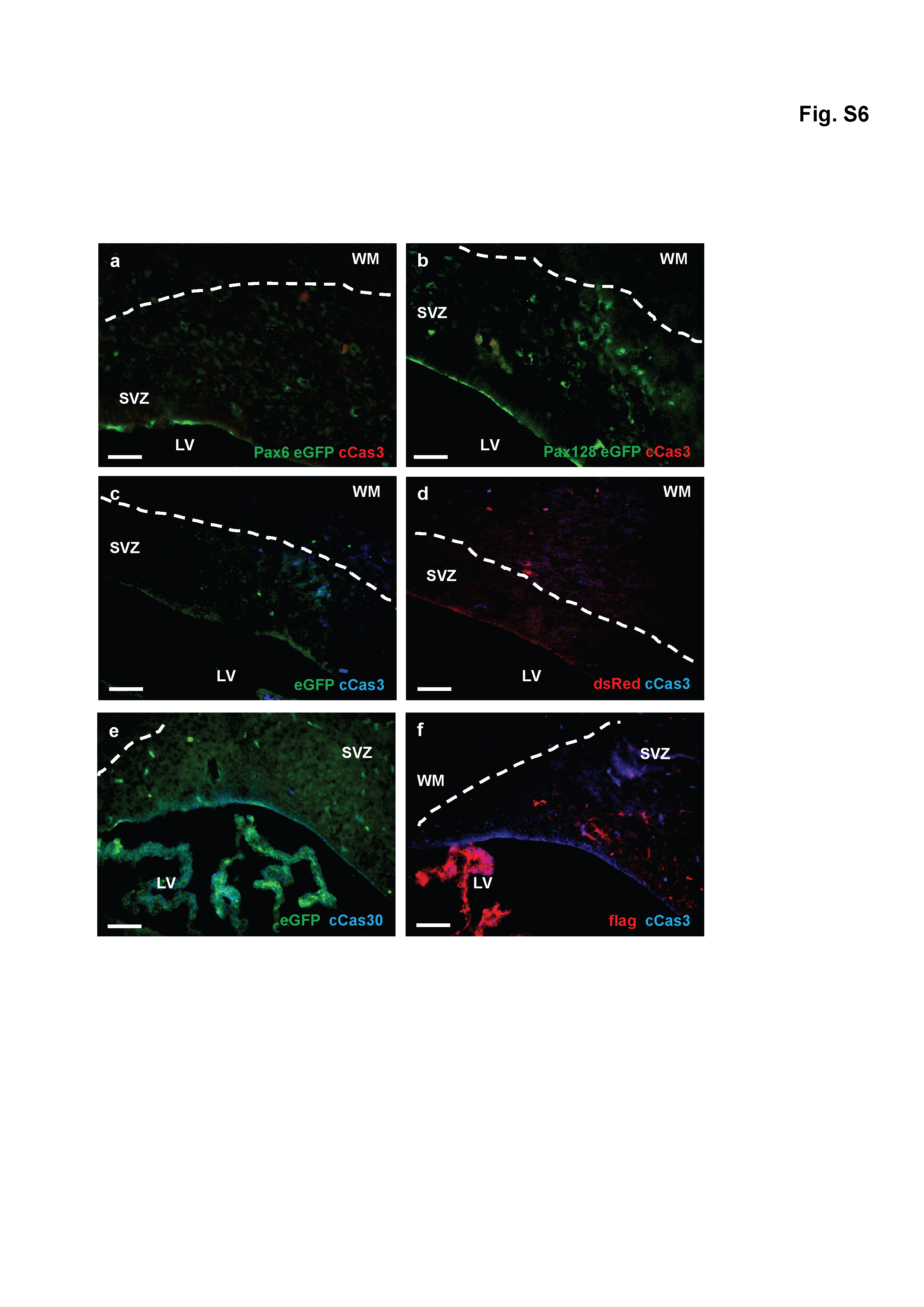

Supplement: Figure S6 — Virus injected brains did not show any distinct cell death. (a, b) Cell death of retrovirus injected brains was determined by immunofluorescence staining with a cleaved caspase 3 antibody (cCas3). eGFP immunostaining in green, cCas3 in red. Micrograph of eGFP and cCas3 immunoreactive cells in a parasagittal plane of postnatal rat SVZ injected with retro pax6-eGFP (a) or pax6(5a) R128C-eGFP virus (b). (c, d) Micrograph of either eGFP (c) or Ds-red (d) and cCas3 immunoreactive cells at parasagittal plane of control lentivirus injected postnatal mouse SVZ. eGFP immunostaining in green, Dsred in red, and cCas3 in Cy5. (e, f) Micrograph of either eGFP (e) or flag (e) and cCas3 immunoreactive cells at parasagittal plane of Pax6-lenti virus injected postnatal mouse SVZ. eGFP immunostaining in green, flag in red, and cCas3 in Cy5. SVZ; subventricular zone, LV; lateral ventricle, WM; white matter. Scale bars, 50 µm. (TIF) [file pone.0020894.s006.tif]
